# The Impact of COVID-19 on Graft Vasculopathy and Postoperative Thromboembolism in CABG Patients: A Prospective Controlled Study

**DOI:** 10.1007/s12012-025-10017-3

**Published:** 2025-06-10

**Authors:** İlknur Şahin, Şebnem Batur, Ahmet Üstündağ, Berk Arapi, Çiğdem Tel Üstünışık, Deniz Göksedef, Suat Nail Ömeroğlu, Gökhan İpek, Ozan Onur Balkanay

**Affiliations:** 1 Basaksehir Cam Ve Sakura City Hospital, Cardiovascular Surgery Clinic, University of Health Sciences, Istanbul, Turkey; 2 Cerrahpasa Medical Faculty, Department of Medical Pathology, Istanbul University-Cerrahpasa, Istanbul, Turkey; 3 Cerrahpasa Medical Faculty, Department of Radiology, Istanbul University-Cerrahpasa, Istanbul, Turkey; 4 Cerrahpasa Medical Faculty, Department of Cardiovascular Surgery, Istanbul University-Cerrahpasa, Istanbul, Turkey

**Keywords:** COVID-19, CABG, Inflammation, Vasculitis

## Abstract

It is known that COVID-19 patients may experience endothelial cell inflammation, apoptosis, dysfunction, and systemic coagulation disorders. In CABG operations, graft patency plays a crucial role in survival and morbidity. Thrombosis and endothelial cell inflammation in grafts can pose challenges for CABG candidates with a history of COVID-19. This study aimed to evaluate the vasculitic effects of a history of COVID-19 among CABG patients. A total of 94 consecutive patients, including 34 with a history of COVID-19 and 60 without, who were scheduled for CABG at our clinic, were included in the study after informed consent was obtained. Patients with a history of COVID-19 underwent surgery at least 4 weeks after the recovery of infection. Thromboembolic events were monitored throughout the hospital stay, and vascular grafts obtained during surgery were pathologically evaluated for signs of vasculitis and inflammation. All COVID-19 (*n* = 34) cases were mild. Statistical analysis revealed no significant difference between the groups regarding vein thrombosis/thrombophlebitis (*p* = 0.626). Additionally, pathological evaluation showed no signs of vasculitis or inflammation. There were also no significant differences in postoperative mortality and morbidity between the two groups (*p* > 0.05). Based on our findings, undergoing CABG surgery after a four-week recovery period appears to be safe for patients with a history of mild COVID-19, at least in terms of early postoperative vascular outcomes.

## Introduction

Coronavirus Disease 2019 (COVID-19), caused by the SARS-CoV-2 virus, varies in severity, ranging from mild respiratory illness to acute respiratory distress syndrome, shock, and multi-organ failure, all associated with an increased risk of mortality [1]. The clinical course of COVID-19 is often accompanied by hyperinflammation and systemic coagulation disorders, which may progress to disseminated intravascular coagulation (DIC) [2]. The combination of local vascular damage, systemic activation of coagulation, and pulmonary thromboinflammation in COVID-19 increases the risk of venous thromboembolism (VTE) and pulmonary artery thrombosis [3]. Additionally, patients with COVID-19 experience endothelial cell inflammation, apoptosis, and dysfunction [4]. Pathological evaluations of lung tissue and other affected organs has revealed evidence of microvascular thrombosis and inflammation. Endothelial cells release proinflammatory cytokines that contribute to the spread of microcirculatory lesions [1]. As a result, dysfunctional endothelial cells become pro-adhesive and prothrombotic [2]. Given these insights into COVID-19-related thrombotic status and vascular pathologies, our study aimed to evaluate the potential vasculitic and thromboembolic effects of a history of COVID-19 among coronary artery bypass grafting (CABG) patients. Furthermore, data on the impact of COVID-19 on postoperative vasculitis and thromboembolism after CABG are limited. Graft patency is crucial for survival and reducing morbidity in CABG. Thrombosis and endothelial cell inflammation within grafts may present challenges for CABG candidates with a history of COVID-19. To address this, our study compared graft patency and postoperative thromboembolic events among patients diagnosed with COVID-19 and those without COVID-19 who underwent CABG surgery for coronary artery disease (CAD). Our prospective controlled study aimed to address the clinically important question: “Is it safe for patients with a history of mild COVID-19 to undergo CABG surgery after a 4-week waiting period, in terms of postoperative graft vasculopathy and thromboembolism?”.

## Materials and Methods

**Fig. 1:**
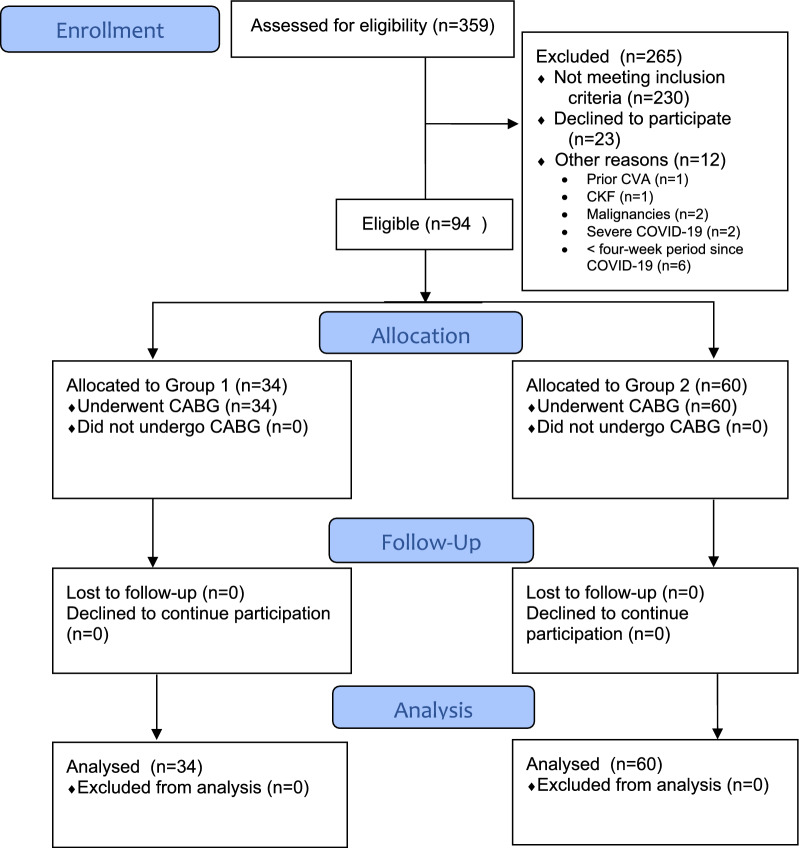
Flow diagram of the study. *CABG* Coronary artery bypass grafting; *CKF* Chronic kidney failure; *COVID-19* Coronavirus Disease 2019; *CVA* Cerebro-vascular accident; Group 1 = Patients with a history of COVID-19; Group 2 = Patients without a history of COVID-19

Between April 2021 and April 2023, a total of 359 patients scheduled for CABG surgery at our clinic due to CAD were assessed for eligibility. Of these, 230 were excluded for not meeting all inclusion criteria, 23 declined to participate, and 12 were excluded for other reasons (Fig. 1). After this assessment, a total of 94 consecutive patients scheduled for CABG surgery due to CAD, with no exclusion criteria, were found eligible and enrolled in the study after obtaining informed consent. The study was conducted in a prospective controlled fashion (Fig. 1). The cohort included patients with a history of COVID-19 (*n* = 34) (confirmed positive RT-PCR test) and those without a history of COVID-19 (*n* = 60). All patients had a history of either asymptomatic or mild COVID-19 infection. None of the patients had a history of hospitalization for COVID-19, nor did any of them experience COVID-19-related complications or require treatment. Standard preoperative and postoperative assessments were conducted, including venous Doppler ultrasonography on postoperative day 7. Samples were collected from the vascular grafts used during surgery (including the IMA and GSV) as well as the aortic punch material for pathological examination. The vaccination status, types, and doses for all patients were recorded.

### Inclusion and Exclusion Criteria

To be eligible for inclusion in the study, patients were required to simultaneously meet all of the following criteria: they must have undergone isolated coronary artery bypass grafting (CABG) without any concomitant procedures such as valve repair or replacement, carotid endarterectomy, or aortic replacement. Only patients scheduled for elective CABG surgery who provided informed consent were included. Additionally, eligible patients were required to be aged 18 years or older, legally capable of providing informed consent, hemodynamically stable during the preoperative period, and have a history of mild or asymptomatic COVID-19 infection. All included patients underwent surgery at our institution between April 2021 and April 2023.

Patients were excluded if they required additional surgical procedures in conjunction with CABG, such as valve replacement, carotid endarterectomy, or ascending aortic replacement, which could influence surgical outcomes. Other exclusion criteria included redo surgeries, the presence of malignancy, chronic kidney disease, or severe heart failure, defined as a left ventricular ejection fraction (LVEF) of ≤ 30%. Patients were also excluded if they had comorbidities that could limit mobilization and thereby increase the risk of deep vein thrombosis, had not completed at least four weeks following COVID-19 infection (to standardize the recovery period and ensure the validity of postoperative outcome evaluations), had a history of severe or hospitalized COVID-19, did not provide informed consent, were younger than 18 years of age (thus not legally eligible to provide consent), underwent emergent or urgent procedures, or were hemodynamically unstable in the preoperative period.

### Statistical Methods

The normality of numerical variables was assessed using visual methods (histogram, Q-Q plot) and analytical methods (Shapiro–Wilk and Kolmogorov–Smirnov tests). Continuous numerical variables are presented as mean ± standard deviation and/or median (interquartile range), while categorical variables are presented as frequency and percentage. The independent samples *T*-test was used for the comparison of two groups with normally distributed data, and the Mann–Whitney U test was used for the comparison of two groups with non-normally distributed data. For dependent group comparisons, the Wilcoxon test was used for the comparison of two measurements, and the Friedman test was used for the comparison of more than two measurements. If necessary, post hoc analysis was performed, and pairwise comparisons were evaluated using the Bonferroni correction. Differences between categorical variables were assessed using Pearson’s chi-square test, and Fisher’s exact test was used when appropriate. A *p*-value of < 0.05 was considered statistically significant. The post hoc power analysis was conducted using the G*Power® Version 3.1 software. All other statistical evaluations were performed using IBM SPSS® Statistics Version 29.0.2.0. Since all patients included in the study were already hospitalized under treatment and closely monitored for CABG, there were no missing data for preoperative, postoperative day 1, and pre-discharge assessments.

### Power Analysis

A post hoc power analysis determined that the statistical power of our study was 74.77%, with a significance level (alpha) of 0.05.

### Preoperative Preparation

In addition to the routine preoperative preparations for patients scheduled for CABG surgery at our clinic [including medical history, physical examination, chest X-ray, electrocardiogram (ECG), echocardiography, routine blood tests such as hemogram, urea, creatinine, alanine transaminase (ALT), aspartate aminotransferase (AST), C-reactive protein (CRP), procalcitonin, prothrombin time (PT), activated partial thromboplastin time (aPTT), international normalized ratio (INR), and anesthesia evaluation], D-dimer, COVID-19 PCR tests and non-contrast computerized tomography (CT) imaging were also performed.

### CABG Indications of Enrolled Patients

- More than 50% stenosis in the left main coronary artery
- Three-vessel coronary artery disease with over 70% stenosis, with or without proximal LAD involvement
- Two-vessel disease with proximal LAD involvement
- Over 70% stenosis in one or more vessels in a patient with significant anginal symptoms despite maximal medical therapy

### Operation

After completing the preoperative preparations, the patients were taken to surgery. Under general anesthesia, a median sternotomy was performed. The GSV and LIMA were prepared as grafts. Following systemic heparinization, cardiopulmonary bypass (CPB) was initiated. The aorta was cross-clamped, and distal anastomoses were performed. The cross-clamp was removed, and a side clamp was applied. Holes were punched in the ascending aorta using a punch tool. Proximal anastomoses were then performed. After the anastomoses were completed, the remaining distal ends of the GSV and LIMA, along with the aortic punch material obtained from the ascending aorta, were placed in formalin and sent to the pathology laboratory for pathological evaluation. After surgery, the patient was transferred to the intensive care unit (ICU).

### Postoperative Period

From the time patients were admitted to the postoperative ICU until their first follow-up on the 10th day after discharge, they were monitored for the development of deep vein thrombosis (DVT) and thromboembolic events.

### Pathological Evaluation

#### Sample Preparation

Vascular specimens that were collected from the vascular grafts used during surgery (including the IMA and GSV) as well as the aortic punch material, were placed in containers containing a 10% formaldehyde solution. Following fixation for 6–12 h, the samples were processed using a closed-system tissue processor (Sakura®-2019) at the Laboratory of the Department of Medical Pathology. The tissue processing protocol included sequential immersion in 10% formalin (2 h, 35 °C, vacuum), alcoholic formalin (1.5 h and 1 h), 95% alcohol (1 h with vacuum and 45 min without vacuum), absolute alcohol (45 min with vacuum and 1 h without vacuum), xylene (1 h with and without vacuum), and paraffin (30 min, 1 h, and 1 h with vacuum), with a total processing time of 12.5 h. Following tissue processing, the specimens were embedded in paraffin blocks. Serial sections with a thickness of 3–4 µm were obtained using a Leica®-2011 microtome for subsequent histological evaluation.

#### Histological Assessment

Prepared sections were stained with Hematoxylin–Eosin (H&E) for general morphological evaluation and with orcein stain to highlight elastic fibers. Each stained section was examined under a light microscope at magnifications of 40 × and 100 × . Vascular specimens were evaluated for the presence of inflammation and vasculitic changes. For each vessel, a minimum of 15 serial sections covering the entire vascular wall were prepared and analyzed. Areas of significant tissue damage were excluded from evaluation. For each staining modality, at least five sections were assessed per vascular specimen. Intimal and medial thickness measurements were obtained from regions where hyperplasia was most pronounced, ensuring that measurements reflected the most pathologically affected areas.

#### Measurement Technique

Histological sections were evaluated by measuring intimal and medial thicknesses at three predefined points around the circumference of each vessel. Consequently, at least 45 measurements were obtained per vascular specimen, incorporating the most affected regions to accurately calculate the mean intimal thickness, mean medial thickness, and the intima-to-media (I/M) ratio. Measurements were performed using an Olympus® Micro DP71 camera integrated with the microscope. Among the serial measurements obtained for each section, the highest values of intimal thickness were selected for analysis to capture the maximum degree of pathological change. Manual measurements were conducted using an ocular micrometer integrated into the microscope eyepiece, enabling precise delineation and measurement of vascular layers. The primary outcomes reported included mean intimal thickness (µm), mean medial thickness (µm), and the I/M ratio, calculated as the ratio of intimal to medial thickness.

## Results

A total of 94 consecutive patients who met all inclusion criteria were included in the study. The patients were divided into two groups: those with a history of COVID-19 (*n* = 34) and those without a history of COVID-19 (*n* = 60) (Fig. 1). Among the patients with a history of COVID-19, 7 (20.6%) were female and 27 (79.4%) were male. In the group without a history of COVID-19, 16 (26.7%) were female and 44 (73.3%) were male. The average age of patients with a history of COVID-19 was 59.9 ± 8.6 years, while the average age of patients without a history of COVID-19 was 57.5 ± 9.5 years. The Body Mass Index (BMI) of patients with a history of COVID-19 was 28 ± 4 kg/m2, while the BMI of those without a history of COVID-19 was 29.1 ± 4.2 kg/m2. There was no statistically significant difference between the groups in terms of age (*p* = 0.233), gender distribution (*p* = 0.51), or BMI (*p* = 0.217). Additionally, there was also no statistically significant difference between the groups in terms of the presence of comorbidities (*p* = 0.594), smoking status (*p* = 0.734), or the number of vessels bypassed during CABG (*p* = 0.2) (Table 1).

**Table 1:** Sociodemographic characteristics of the groups

Characteristic	Group 1 (*n* = 34)	Group 2 (*n* = 60)	*p*-value
*Gender, n (%)*			0.51
Female	7 (20.6)	16 (26.7)	
Male	27 (79.4)	44 (73.3)	
Average Age (mean ± SD)	59.9 ± 8.6	57.5 ± 9.5	0.233
Body Mass Index (mean ± SD)	28 ± 4	29.1 ± 4.2	0.217
*Type of Vaccination, n (%)*			0.629
None	9 (26.5)	14 (23.3)	
Biontech®	11 (32.4)	23 (38.3)	
Sinovac®	3 (8.8)	6 (10)	
Biontech® + Sinovac® (B + S)	9 (26.5)	17 (28.3)	
Biontech® + Sinovac® + Turkovac® (B + S + T)	1 (2.9)	0 (0)	
Biontech® + Turkovac® (B + T)	1 (2.9)	0 (0)	
*Comorbidities, n (%)*			0.594
None	6 (17.6)	16 (26.7)	
1 comorbidity	9 (26.5)	13 (21.7)	
> 1 comorbidity	19 (55.9)	31 (51.7)	
*Smoking Status, n (%)*			0.734
Smoker	25 (73.5)	46 (76.7)	
Non-smoker	9 (26.5)	14 (23.3)	
Number of vessels bypassed (median, IQR)	4 (3–4)	3.5 (3–4)	0.2
ICU Stay Duration (days) (mean ± SD)	2.9 ± 2.2	3.7 ± 4.8	0.582
Hospital Stay Duration (days) (mean ± SD)	7 ± 2.4	8.2 ± 5.8	0.389
In-hospital Mortality, n (%)(mean ± SD)	3 (8.8)	2 (3.3)	0.348

*Comorbidities* Carotid artery disease, peripheral artery disease, diabetes mellitus; *ICU* Intensive Care Unit; *IQR* Interquartile Range; *SD* Standard Deviation

Group 1 = Patients with a history of COVID-19; Group 2 = Patients without a history of COVID-19

There was no statistically significant difference between the groups regarding the distribution of the types of vaccines received by the patients (*p* = 0.629) (Table 1).

Intensive care unit and hospital stay durations, as well as mortality rates, were compared between the groups, and no statistically significant differences were found (*p* > 0.05) (Table 1).

**Fig. 2:**
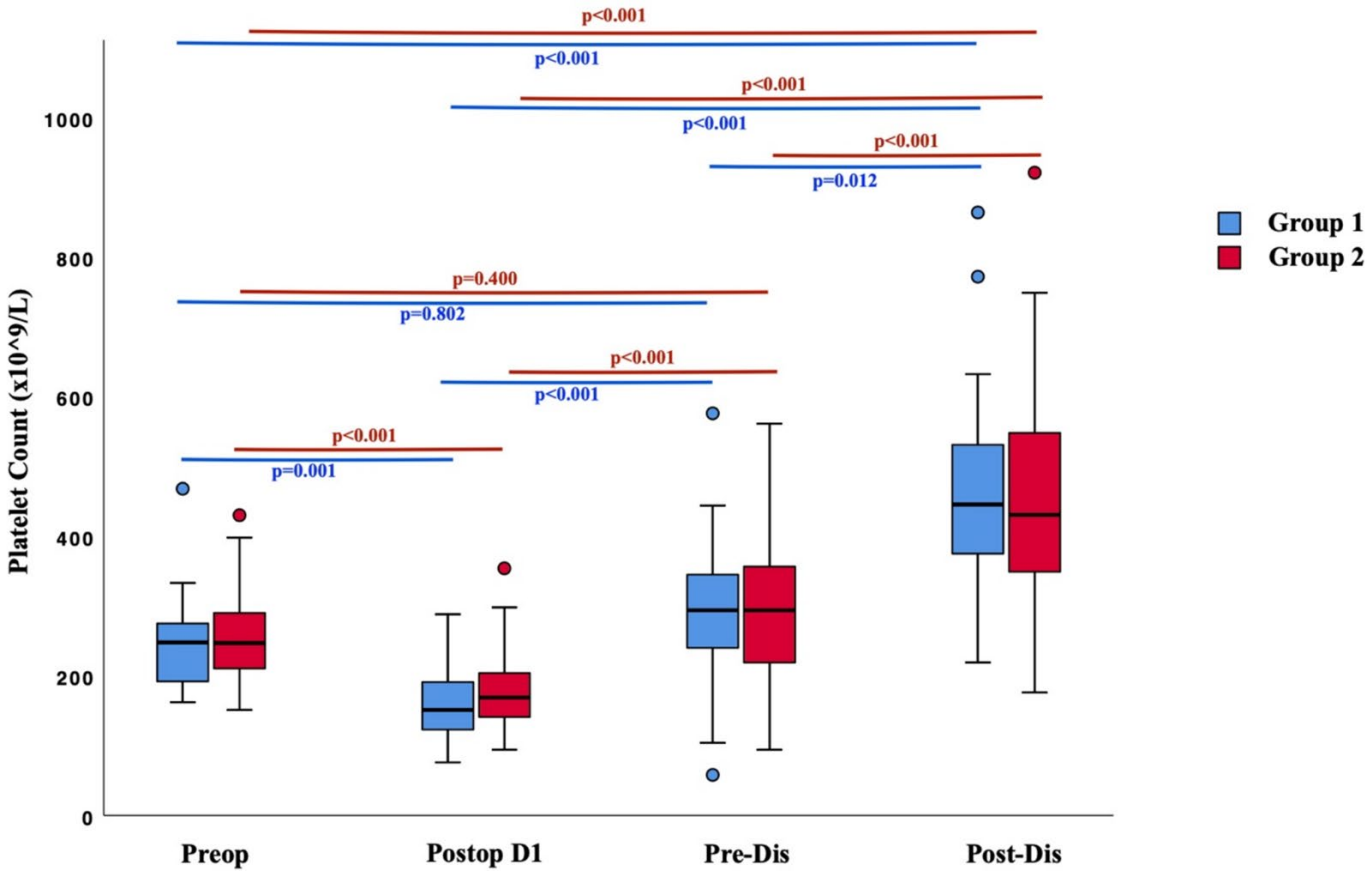
Change in platelet count for both groups. *Pre-Dis* Pre-Discharge; *Preop* Preoperative; *Postop* Postoperative; *Post-Dis* Post-Discharge; Group 1 = Patients with a history of COVID-19; Group 2 = Patients without a history of COVID-19

The changes in platelet counts before surgery, on the first postoperative day, before discharge, and after discharge were examined. The change in platelet count was found to be statistically significant in both groups (*p* < 0.001) (Table 2; Figs. 2). Post hoc analyses were conducted to determine between which two measurements the statistical significance occurred. In the group with a history of COVID-19, there was no significant difference between the preoperative and pre-discharge platelet levels, while statistically significant differences were observed between all other measurements (*p* < 0.05). Similarly, in the group without a history of COVID-19, there was no significant difference between the preoperative and pre-discharge platelet levels, while statistically significant differences were found between all other measurements (*p* < 0.05) (Fig. 2).

**Table 2:** Comparison of platelet levels between groups

Platelet Count (× 10⁹/L)	Preop	Postop D1	Pre-Dis	Post-Dis	*P*
Group 1 (mean ± SD)	245.6 ± 60.1	157.8 ± 45.3	292.4 ± 100.9	455.5 ± 144.3	< 0.001*
Group 2 (mean ± SD)	253.8 ± 54.6	177.8 ± 51.3	296.3 ± 96.9	451.4 ± 146.6	< 0.001*
*P*	0.341	0.063	0.858	0.863	

*ICU* Intensive Care Unit; *Pre-Dis* Pre-Discharge; *Preop* Preoperative; *Postop* Postoperative; *Post-Dis* Post-Discharge; *SD* Standard Deviation

Group 1 = Patients with a history of COVID-19; Group 2 = Patients without a history of COVID-19

*statistically significant (*p* < 0.05)

**Fig. 3:**
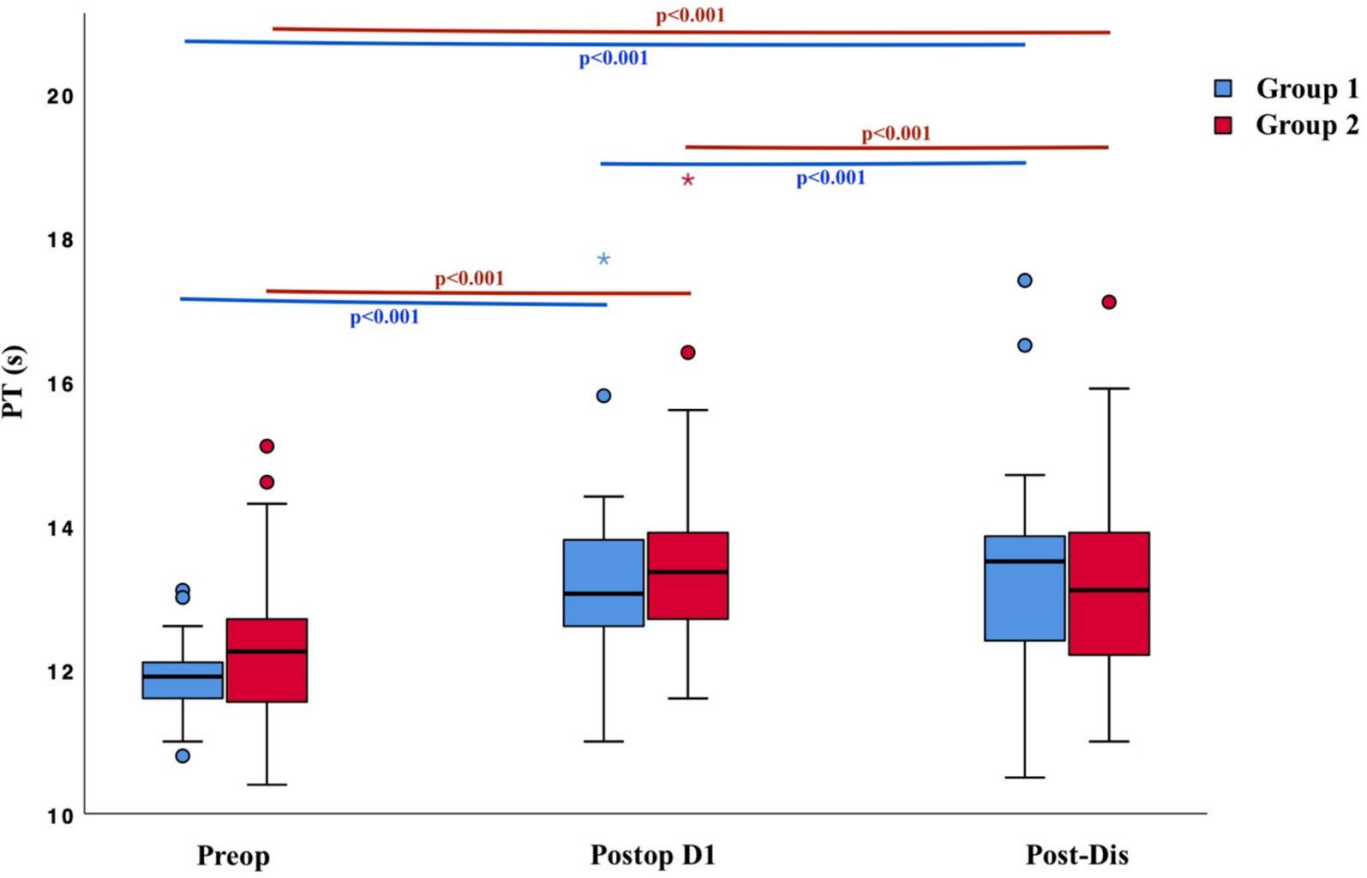
Change in PT Levels for both Groups. *Preop* Preoperative; *Postop* Postoperative; *Post-Dis* Post-Discharge; Group 1 = Patients with a history of COVID-19; Group 2 = Patients without a history of COVID-19

The changes in PT, aPTT, and INR levels before surgery, on the first postoperative day, and after discharge were examined within each group. The change in PT was found to be statistically significant in both the group with a history of COVID-19 and the group without a history of COVID-19 (*p* < 0.001 and *p* < 0.001) (Fig. 3). In the post hoc analyses, the differences between preoperative PT and both postoperative day 1 PT and post-discharge PT were statistically significant in the group with a history of COVID-19 (*p* < 0.001 and *p* < 0.001). Similarly, in the post hoc analysis for PT in the group without a history of COVID-19, the differences between preoperative PT and both postoperative day 1 PT and post-discharge PT were statistically significant (*p* < 0.001 and *p* < 0.001). In the group with a history of COVID-19, there was no statistically significant difference between the preoperative, postoperative day 1, and post-discharge aPTT values (*p* > 0.05). However, the change in aPTT was statistically significant in the group without a history of COVID-19 (*p* = 0.007). In the post hoc analysis, the difference between the aPTT values measured on postoperative day 1 and after discharge was statistically significant (*p* = 0.009). The changes in INR levels before surgery, on the first postoperative day, and after discharge were found to be statistically significant in both groups (*p* < 0.001 and *p* < 0.001). In the post hoc analyses, there was a statistically significant difference between the preoperative INR and both postoperative day 1 INR and post-discharge INR in both groups (Group with a history of COVID-19: *p* < 0.001, *p* < 0.001; Group without a history of COVID-19: *p* < 0.001, *p* < 0.001). However, no statistically significant change was observed between the INR values on postoperative day 1 and after discharge in either group (*p* > 0.05). There were no statistically significant differences between the two groups in preoperative and postoperative serum chemistry parameters, including CRP, urea, creatinine, ALT, and AST (*p* > 0.05). The statistical analysis results of the PT, aPTT, and INR levels and the changes between measurements in the groups are presented in Table 3.

**Table 3:** Comparison of changes in coagulation, renal, and liver function tests within and between groups

	Group 1‡			*P*	Group 2‡			*P*
	Preop (Mean ± SD)	Postop Day 1 (Mean ± SD)	Post-Discharge (Mean ± SD)		Preop (Mean ± SD)	Postop Day 1 (Mean ± SD)	Post-Discharge (Mean ± SD)	
PT (s)	11.9 ± 0.5	13.4 ± 1.3	13.3 ± 1.3	< 0.001*	12.2 ± 0.9	13.5 ± 1.2	13.3 ± 1.1	< 0.001*
aPTT (s	25.7 ± 3	34.5 ± 3.9	24.7 ± 2.8	0.106	29.1 ± 16.6	32.7 ± 2.9	23.9 ± 2.7	0.007*
INR	1 ± 0.06	1.15 ± 0.1	1.13 ± 0.12	< 0.001*	1.03 ± 0.08	1.15 ± 0.11	1.13 ± 0.11	< 0.001*
CRP (mg/L)	16.26 ± 22.85	59.62 ± 43.26		< 0.001*	10.8 ± 13.22	53.22 ± 25.9		< 0.001*
Urea (mg/dL)	31.5 ± 9.2	31.18 ± 8.05		0.897	35.8 ± 17.66	35.4 ± 15.85		0.628
Creatinine (mg/dL)	0.91 ± 0.19	0.94 ± 0.27		0.681	0.94 ± 0.36	0.96 ± 0.44		0.952
ALT (U/L)	24.1 ± 10.82	36.07 ± 22.77		0.002*	26.61 ± 17.22	30.38 ± 12.7		0.023*
AST (U/L)	31.52 ± 29.92	63.54 ± 34.81		< 0.001*	24.95 ± 12.1	65.65 ± 39.9		< 0.001*

*ALT* Alanine Aminotransferase; *aPTT* Activated Partial Thromboplastin Time; *AST* Aspartate Aminotransferase; *CRP* C-Reactive Protein; *INR* International Normalized Ratio; *Preop* Preoperative; *Postop* Postoperative; *PT* Prothrombin Time; *SD* Standard Deviation

Group 1 = Patients with a history of COVID-19; Group 2 = Patients without a history of COVID-19

‡There were no statistically significant differences between the two groups in preoperative and postoperative serum chemistry parameters (*p* > 0.05)

*statistically significant (*p* < 0.05)

The changes in CRP, urea, creatinine, ALT, and AST levels before and after surgery were statistically analyzed within each group. The CRP levels in both groups increased significantly after surgery (median 8 mg/L vs. 49.48 mg/L; 3.95 mg/L vs. 50.79 mg/L, respectively) (*p* < 0.001 and *p* < 0.001). ALT and AST levels also increased significantly after surgery in both the group with a history of COVID-19 and the group without a history of COVID-19 (for the COVID-19 history group: *p* = 0.002, *p* < 0.001, respectively; for the group without a COVID-19 history: *p* = 0.023, *p* < 0.001, respectively). The changes in urea and creatinine levels measured before and after surgery were not statistically significant in either group (*p* > 0.05). The preoperative (0.41 ± 0.29) and postoperative first-day (1.95 ± 2.00) D-dimer values differed significantly (*p* < 0.001); however, no significant difference was observed between the groups. The preoperative and postoperative values of CRP, urea, creatinine, ALT, and AST, as well as the statistical analysis results for each group, are presented in Table 3.

In the group with a history of COVID-19, thoracic CT scans revealed evidence of mild COVID-19 involvement in 67.6% (*n* = 23) of the group, while no involvement was observed in 32.4% (*n* = 11) of the group. An additional analysis comparing the thoracic CT scan-positive and scan-negative groups in terms of saphenous vein intima/media ratio values revealed no statistically significant difference between the groups (scan-positive group: 0.44 ± 0.5 vs. scan-negative group: 0.53 ± 0.6; *p* = 0.518).

During the preoperative period, venous Doppler ultrasound examinations revealed no pathological findings in either group. However, on the 7th postoperative day, venous Doppler ultrasound revealed thrombophlebitis in 1 patient (3%) in the group with a history of COVID-19, while in the group without a history of COVID-19, thrombosis in the left popliteal vein was detected in 1 patient (1.7%) and thrombophlebitis was detected in 2 patients (3.4%). Statistical analysis revealed no significant difference between the groups regarding vein thrombosis/thrombophlebitis (*p* = 0.626). A multivariate analysis was conducted to evaluate the possible variables that could affect thromboembolic events. This analysis showed no significant variables (Table 4).

**Table 4:** Multivariate analysis of thromboembolic events

Variable	OR	(95% CI)	*P*-value
Gender (male)	2.417	0.257–22.703	0.440
History of Covid-19	1.723	0.152–19.560	0.661
Age (each year)	1.035	0.920–1.164	0.565
BMI	1.023	0.791–1.323	0.863
HT	0.596	0.048–7.465	0.689
DM	0.340	0.030–3.870	0.385

*BMI* body mass index; *CI* confidence interval; *DM* diabetes mellitus; *HT* hypertension; *OR* odds ratio

**Fig. 4:**
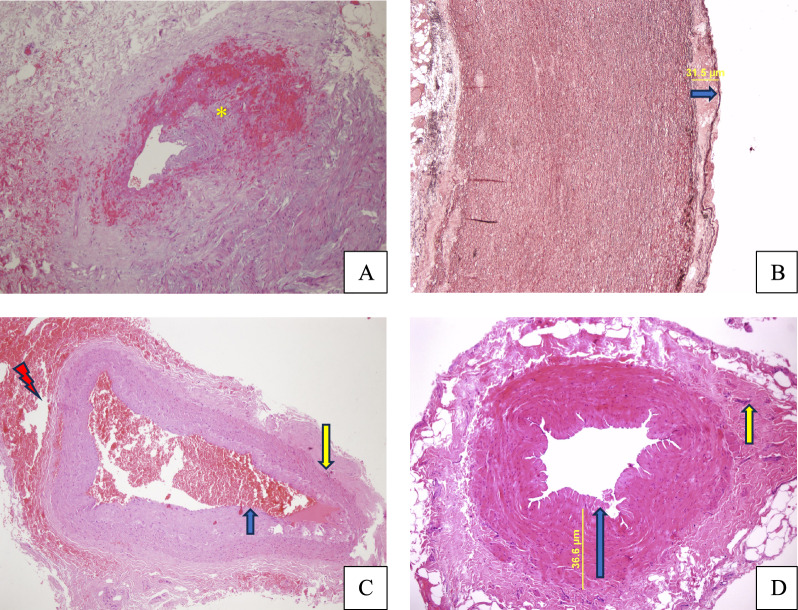
Images from the Light Microscopic Evaluation of Tissues. **A** LIMA (Left Internal Mammary Artery)—Fresh thrombus in the vessel lumen (yellow asterisk) (Hematoxylin & Eosin, H&E, × 100). **B** Aorta—Myointimal proliferation in the intima (blue arrow), loss of elastic fibers in the media (Orcein, × 100). **C** LIMA—Myointimal proliferation in the intima (blue arrow), mild fibrosis in the adventitia (yellow arrow), and signs of fresh hemorrhage (red lightning) (H&E, × 100). **D** Saphenous Vein—Mild proliferation in the intima (blue arrow), thickening in the media, mild fibrosis in the adventitia (yellow arrow) (H&E, × 100)

The vascular structures were evaluated under a light microscope. The vascular materials were examined for signs of inflammation and vasculitis. No findings indicative of inflammation or vasculitis were observed in the saphenous vein, LIMA, or aorta tissues during the light microscopic evaluation. Myointimal/intimal hyperplasia consistent with an atherosclerotic process was found in all vascular structures. Acute thrombosis was observed in the LIMA lumen of two patients. Light microscopic images of the tissues are shown in Fig. 4 below. The intimal and medial thicknesses of the saphenous vein and aorta tissues were measured, and the ratios were calculated. The saphenous intima, saphenous media, saphenous intima/media ratio, aortic intima, aortic media, and aortic intima/media ratio were compared among the patients, and no statistically significant differences were found between the groups (*p* > 0.05) (Table 5).

**Table 5:** Comparison of intima and media thickness and intima/media ratio of saphenous vein and aorta

	Group 1 (*n* = 34)		Group 2 (*n* = 60)		
	n*	(Mean ± SD)	n*	(Mean ± SD)	*P*
SV Intima Thickness	27	22.03 ± 20.49	57	14.13 ± 10.09	0.189
SV Media Thickness	27	42.59 ± 30.35	57	49.99 ± 33.85	0.178
SV I/M Ratio	27	0.72 ± 0.75	57	0.41 ± 0.4	0.061
Ao Intima Thickness	14	16 ± 13.64	43	9.68 ± 9	0.214
Ao Media Thickness	14	155.11 ± 63.01	43	134.76 ± 44.24	0.326
Ao I/M Ratio	14	0.12 ± 0.13	43	0.07 ± 0.07	0.349

Group 1 = Patients with a history of COVID-19; Group 2 = Patients without a history of COVID-19; *Ao* Aorta; *I/M* Intima/Media; *SD* Standard Deviation; *SV* Saphenous Vein. *For each patient, a minimum of 45 measurements were obtained per vascular specimen, specifically targeting the most affected regions, and a mean value was calculated accordingly

## Discussion

The primary clinical concern for patients with a history of COVID-19 undergoing CABG is the potential increase in graft vasculopathy and thromboembolism. Beyond the inflammatory response triggered by CPB support during CABG, COVID-19 infection may be associated with coagulopathy, endothelial damage, and vasculitis, all of which could contribute to postoperative graft vasculopathy and thromboembolism. Although this is a clinically important issue, the existing literature on CABG and its outcomes in patients with history of COVID-19 infection is limited. In our study, a total of 94 patients who underwent CABG surgery, including those with and without a history of COVID-19 infection, were examined. Patients with and without a history of COVID-19 prior to surgery were compared in terms of baseline characteristics, postoperative complications, and outcomes. The demographic characteristics were similar in both groups, and no statistically significant difference was found in terms of baseline characteristics. The demographic and risk factor profiles were consistent with international studies conducted on CABG patients [5]. The similarities between the groups allowed for a clearer focus on the effects of COVID-19 in terms of outcomes. Following the recommendation that patients scheduled for elective surgery should wait at least 4 weeks after recovering from mild respiratory symptoms or asymptomatic COVID-19, we performed surgery on our patients 4 weeks after they had recovered from COVID-19 [6]. The waiting period for optimal recovery was standardized to ensure a uniform comparison. None of the patients with a history of COVID-19 had been hospitalized due to the infection. All patients who had recovered COVID-19 experienced the disease in a mild form. Our study aimed to investigate and better understand the effects of a history of COVID-19, even in its mild form, on CABG outcomes, specifically regarding postoperative graft vasculopathy and thromboembolism.

### COVID-19 Coagulopathy and Endothelial Damage

Reports related to COVID-19 have described hypercoagulability and a thrombotic tendency [7]. Recent reports of venous and arterial thrombotic events in patients treated in ICUs indicate that rates can reach up to 30% even with pharmacological thromboprophylaxis, and these thrombotic events are associated with a 5.4-fold higher risk of mortality [8]. Postmortem evaluations of COVID-19 patients have shown severe endothelial damage with cellular death/apoptosis, thrombosis in the lungs, and small to medium-sized pulmonary vessels, along with intracellular presence of the virus. Clotting and vascular damage in the alveolar capillaries have also been confirmed, and these changes are more pronounced in COVID-19 compared to lung damage caused by influenza [9]. Although changes in fibrinogen and D-dimer levels were more pronounced, prolonged PT were observed in COVID-19 patients compared to healthy controls [7]. Prolonged PT was observed in COVID-19 patients who did not survive and those in ICU. Mildly prolonged aPTT has also been reported [10, 11]. It may not be entirely accurate to attribute prolonged aPTT solely to COVID-19, as there are various causes for this, such as heparin use, the presence of lupus anticoagulant, and elevated C-reactive protein levels [12–14]. In our study, prolonged PT was observed in both the group with a history of COVID-19 and the group without, and this was found to be statistically significant. There was no statistically significant difference in aPTT values measured preoperatively, on the first postoperative day, and before discharge in the group with a history of COVID-19. However, in the group without a history of COVID-19, the increase in aPTT values measured on the first postoperative day and before discharge was statistically significant, but it was not considered significant concerning the association with COVID-19. The findings of our study indicate that, in CABG patients, a history of mild COVID-19 infection did not negatively affect coagulation status in terms of PT and aPTT.

Thromboembolic complications are a distinctive feature of COVID-19, potentially leading to death even in asymptomatic cases [15]. The novel coronavirus SARS-CoV-2 triggers acute inflammation with hypercoagulability, platelet activation, and endothelial dysfunction [16]. In COVID-19-associated coagulopathy, patients often present initially with increased fibrinogen levels, elevated D-dimers, but with only minor changes in PT and platelet count compared to acute bacterial sepsis, which can lead to thrombocytopenia, prolonged prothrombin times, and decreased antithrombin levels [8, 9, 15–18]. Additionally, it is known that inflammatory cytokine levels rise in COVID-19, and the excessive production of these cytokines can induce hemophagocytic lymphohistiocytosis (HLH)/macrophage activation syndrome (MAS), which may result in thrombotic clotting disorders [19]. Additionally, the incidence of thrombocytopenia in COVID-19 patients is relatively low [20]. There are many studies published on this topic with conflicting evidence regarding whether thrombocytopenia can be used as a prognostic biomarker [21]. Several mechanisms responsible for thrombocytopenia and thrombocytosis require further investigation [20]. In our study, the change in platelet count was found to be statistically significant in both the group with a history of COVID-19 and the group without. The relationship between this increase in platelet count and vaccinations was examined, and no association was found. It was not considered that the increase in platelet count was related to the COVID-19 disease.

A significant feature of COVID-19-related coagulopathy is microvascular endothelial damage in the pulmonary circulation and other vascular beds. As SARS-CoV-2 directly infects vascular endothelial cells, causing cellular damage and apoptosis, the antithrombotic activity of the luminal surface is significantly reduced [22]. Direct viral infection or immune-mediated uptake of immune cells by the endothelium may lead to widespread endothelial dysfunction associated with apoptosis [23]. Furthermore, postmortem biopsies of patients who died due to COVID-19 revealed macro- and microvascular thrombosis in the arteries, veins, arterioles, capillaries, and venules of all major organs [24]. In this postmortem analysis, mononuclear cell infiltrations along the vascular intima of many vessels were also reported. This finding suggests that the virus can invade the human vascular system and potentially cause vasculitis. Another study highlighted that the progression from type 2T-helper cell immune response to type 3 hypersensitivity plays a role in the pathophysiology of COVID-19-induced vasculitis. It was reported that the accumulation of immune complexes within the vascular walls leads to a more severe inflammatory reaction, with interleukin-6 identified as the key myokine in this scenario [25]. The microcirculatory dysfunction associated with both alveolar damage and thrombosis in COVID-19 contributes to respiratory dysfunction. Normal endothelial function involves regulating vascular tone, permeability, cell adhesion, and anticoagulation. Endothelial damage leads to procoagulant changes in the vessel lumen, immunothrombosis formation, and circulatory disorders in organs. Due to the underlying mechanisms of COVID-19, including an increased risk of endothelial damage and coagulopathy, there is concern about a potentially higher risk of graft failure after CABG. As is well known, the most critical factor affecting venous graft patency after coronary artery bypass surgery is intimal hyperplasia. The maintenance of venous graft patency depends on several factors, including the quality, size, and diameter of the saphenous vein; the size and diameter of the target coronary artery; surgical skill; intraoperative use of the venous graft material; and (perioperative and postoperative) medical management [26–28]. These changes were associated with thickening of the intimal layer in both vessels, despite the absence of inflammation. A 2000-fold increase in the intima/media ratio was observed in vascular patches [29]. Therefore, vascular grafts, including GSV and LIMA specimens, were evaluated in our study for myointimal hyperplasia, intima/media ratio, and thickness. During these measurements, the site of maximum intimal thickening was selected due to its methodological and clinical advantages. This approach enables the identification of the most advanced pathological changes, increases sensitivity for detecting early or subtle lesions, enhances interobserver reproducibility, and demonstrates a stronger correlation with adverse clinical outcomes such as graft failure and vascular restenosis. Moreover, focusing on the point of maximum thickness reduces the risk of underestimating disease severity, thereby improving the prognostic utility of pathological assessments. Previous studies, including evidence from the Framingham Offspring Study, have confirmed that measurements based on maximum intimal thickness outperform mean values in predicting cardiovascular risk, further validating the methodological approach employed in our study [30]. Myointimal hyperplasia was observed in all vascular tissues (aorta, LIMA, and saphenous vein) obtained from patients, regardless of COVID-19 history. The saphenous intima, saphenous media, saphenous intima/media ratio, aortic intima, aortic media, and aortic intima/media ratio were compared among the patients, and no statistically significant differences were found between the groups. The presence of such hyperplasia in venous structures even before surgery suggests that the risk factors for atherosclerosis and coronary artery disease may also affect venous structures as much as arterial structures. Given the role of intimal hyperplasia in the failure of graft patency after CABG, the presence of myointimal hyperplasia in prepared grafts before anastomosis is noteworthy. However, our study did not find an increase in vascular graft pathologies related to a history of COVID-19 infection.

### COVID-19 and Vasculitis

Some pathophysiological observations related to thrombosis in small to medium-sized arteries in COVID-19 are intriguing [31]. This complication can be associated with two main processes. The first is acute endotheliitis, in which endothelial cells harboring virions undergo accelerated apoptosis and lymphocytic endotheliitis, resulting in an inflammatory/prothrombotic environment due to leakage from neutrophils and mononuclear cells [32]. This process is associated with a cell-mediated immune response. Secondly, in the later stages of the disease, peri/panarteritis develops in the same arteries due to prolonged infiltration by the same inflammatory cells. This is followed by accelerated karyolysis, accumulation of apoptotic bodies, caspase protein granules, and fibrinoid material. Varga and colleagues have demonstrated endothelial cell involvement and endotheliitis in vascular beds [32]. Histology and electron microscopy have shown the accumulation of inflammatory cells and viral inclusions within the vascular endothelium of the heart, small intestine, kidneys, and lungs. Endotheliitis within dermal vesicles and lymphocytic infiltration of arterioles, along with microthrombosis of papillary dermal capillaries, are common findings. While thrombosis often accompanies vasculitis, obliterative arteriolitis also contributes to tissue damage [33]. This study suggests that endothelial damage is associated with the severity of COVID-19 [34]. In light of this literature, we suspected and evaluated underlying vasculitis or DVT in patients with a history of COVID-19, as this could increase postoperative thromboembolism in CABG patients. Therefore, venous Doppler ultrasound evaluations were performed preoperatively and on the 7th postoperative day. These evaluations showed that none of the patients had a history of vasculitis or DVT, and no clinically significant thromboembolic events occurred during follow-up. Contrary to our findings, Varga and colleagues have pathologically demonstrated endothelial cell involvement and endotheliitis in the vascular beds. Inflammatory cells and viral inclusions were observed in the vascular endothelium of the heart, small intestine, kidneys, and lungs, using histology and electron microscopy [32]. In our study, light microscopic examinations did not reveal any findings suggestive of inflammatory cell accumulation or inflammation in vascular tissues. Independent of the study’s objective, acute thrombosis was observed at the microvascular level in the LIMA lumens of two patients without a history of COVID-19. This condition, which was not observed macroscopically, may affect graft patency. Other than this, no differences were observed between tissue samples from the two patient groups. An important point to highlight in explaining these contrary findings is that previous studies on endothelial damage and vasculitis have generally been conducted in patients with severe disease requiring hospitalization. As a result, research on thromboembolism and vasculitis in patients who experienced asymptomatic or mild COVID-19 is limited. Unlike previous studies, our study compared CABG patients without a history of COVID-19 infection to those with mild COVID-19 infection, none of whom required hospitalization for COVID-19. As a result, our findings indicate that CABG can be safely performed in patients who have recovered from mild COVID-19, with outcomes comparable to those of patients without a history of COVID-19 in terms of thromboembolism and vasculitis.

### Long-Term Effects of COVID-19 on the Vascular System

Data on the long-term effects of COVID-19 on vascular function are even scarcer than those on the heart. During the pandemic, researchers demonstrated that acute COVID-19 is associated with severe pulmonary and extrapulmonary vascular inflammation at both macrovascular and microvascular levels [9]. Additionally, pulmonary and extrapulmonary thromboembolism are common complications that define both the initial and potentially long-term outcomes of COVID-19 [35]. Cytokines such as TNF-α and IL-1β are well known for their proinflammatory effects on the endothelium and may play a significant role in vascular dysfunction in COVID-19 [36]. In a study conducted 68 days after confirmed SARS-CoV-2 infection involving 50 patients, the analysis of acute phase markers, endothelial cell activation, and thrombin production showed sustained endothelial cell activation for up to 10 weeks following acute SARS-CoV-2 infection [37]. The data also suggested that endothelial dysfunction occurred independently of the ongoing acute phase response but was associated with increased thrombin production. The authors further hypothesized that the shedding of thrombin from endothelial cells may play a role in modulating the loss of normal endothelial cell quiescence [37]. Another study investigated the potential effects of SARS-CoV-2 on the systemic vascular system in the arms and legs of 20 young adults. Using a cross-sectional design, two studies assessed vascular function 3–4 weeks after SARS-CoV-2 infection using Doppler ultrasound to measure flow-mediated dilation in the arm and single passive limb movement in the leg. In addition, carotid-femoral pulse wave velocity, an indicator of arterial stiffness, was evaluated. The results showed significantly lower systemic vascular function and greater arterial stiffness in participants who tested positive for SARS-CoV-2 compared to controls. These studies included both male and female participants but found no gender-specific effects [38]. In a similar study design but with a longer follow-up period (3 months after COVID-19 diagnosis), 16 young adults were evaluated for brachial flow-mediated dilation, cerebral vasodilator function, and arterial stiffness. Of these participants, eight were still symptomatic, while the others no longer showed COVID-19 symptoms. Subsequent analyses revealed that peripheral macrovascular and microvascular vasodilation was significantly reduced in young adults who were still symptomatic, whereas asymptomatic participants had similar vascular function compared to controls. The term ‘long COVID’ is used to describe vascular involvement that develops more than 12 weeks after the acute phase of COVID-19 infection. Within this definition, vascular involvement may include inflammation of the vascular endothelium, the development of endothelial dysfunction, and a clinical picture characterized by the progression of previously affected atherosclerotic plaque regions. Several studies have reported that such changes may lead to thromboembolic arterial or venous complications [39]. In this context, treatment strategies have even been proposed to prevent long-term effects associated with prolonged humoral immune responses in certain patients [40]. Additionally, since ongoing vascular endothelial injury—triggering platelet aggregation and coagulation—plays a key role in the pathophysiology of ‘long COVID,’ the early initiation of anticoagulant therapy is currently under discussion [41]. Since our study only evaluated the 4-week period after COVID-19 recovery and the early postoperative period of CABG, including graft evaluations and early outcomes, we cannot make definitive conclusions regarding the long-term effects of COVID-19, despite the similar findings between groups. More comprehensive studies are needed to understand the long-term effects of COVID-19 on the vascular system.

### Cardiopulmonary Bypass and Inflammation

Several studies have shown that CABG triggers a complex prothrombotic and proinflammatory response that peaks from the end of CPB to the early hours following protamine administration, depending on the biomarkers considered. The activation of plasmatic (contact, intrinsic coagulation, extrinsic coagulation, complement, and fibrinolytic systems) and cellular (platelets, neutrophils, monocytes, endothelial cells, and lymphocytes) blood components occurs at two distinct times and mechanisms: upon contact with the non-endothelialized foreign surfaces of the surgical field, CPB circuit, or cell saver device. It is now well-established that contact with the surgical field significantly contributes to the initial activation of the hemostatic system, which ultimately leads to thrombin formation [42, 43]. Moreover, a burst of complement (still under debate) is expected during aortic cross-clamp removal, with a burst occurring during protamine administration. All these events stimulate neutrophils, monocytes, and endothelial cells to release proinflammatory and anti-inflammatory cytokines, promoting and amplifying cell-derived inflammation [44–50]. Consequently, in patients undergoing cardiac surgery with cardiopulmonary bypass, a systemic inflammatory response is triggered by a combination of surgical trauma, activation of blood components in the extracorporeal circulation, ischemia/reperfusion injury, endotoxin release, and activation of immune cells [51–54]. However, beyond the inflammatory response triggered by CPB, data on the impact of COVID-19 on patients undergoing open-heart surgery is limited. The prognosis is generally poor in patients who recover COVID-19 and experience acute coronary syndrome (ACS). Continued myocardial ischemia or necrosis in these patients may lead to decreased cardiac function, making heart failure more likely, which can cause sudden clinical deterioration. In patients with underlying heart disease and heart failure, COVID-19 infection may exacerbate the clinical condition and potentially lead to death [55]. Previous studies have shown high morbidity and mortality in groups of patients with COVID-19 who underwent or were recovering from cardiac surgery [56–60]. A recent study presented eighteen-month follow-up data for a patient with COVID-19 who underwent CABG surgery on a beating heart and was subsequently discharged. The authors suggested that avoiding CPB might result in better outcomes, considering factors such as systemic inflammatory response, damage to red blood cells and platelets, increased catecholamine levels, complement activation, protein denaturation, and increased extracellular fluid [61]. Cardiopulmonary bypass may cause harmful effects on the heart, kidneys, liver, and lungs due to non-pulsatile flow, hypothermia, duration of CPB, hypoperfusion, and microemboli causing stroke, all of which can be exacerbated by the underlying disease. A new study involving twelve patients (with an in-hospital mortality rate of 16.67%) suggested that cardiac surgery could be performed safely in a similar subgroup of patients, particularly in those with asymptomatic to mild COVID-19 infection [62]. Some studies suggest that COVID-19 infection may exacerbate the post-inflammatory state following cardiopulmonary bypass (CPB), potentially increasing the risk of adverse postoperative outcomes [63]. However, in contrast to some previous literature suggesting that CPB should be avoided for better outcomes, all CABG operations in our study were conducted using CPB. In addition to finding CPB a more secure technique for achieving good outcomes in CABG, there were other reasons for selecting CPB in our study patients. First, all patients with a history of COVID-19 had fully recovered. Second, these patients had a history of mild COVID-19 that did not require hospitalization. Third, most patients included in our study had multivessel disease, which is not primarily suitable for a minimally invasive off-pump CABG approach. Additionally, comparing the groups' ICU and hospital stay durations and mortality rates revealed no statistically significant differences. In the COVID-19 group, three patients (8.8%) died, while in the control group, two patients (3.3%) died; however, this difference was not statistically significant. Regarding the causes of mortality, all deaths in both groups were due to cardiac causes. Three patients in the COVID-19 group and one patient in the control group were discharged from surgery with high-dose inotropic support and intra-aortic balloon pump (IABP) support and required ECMO support during ICU follow-up. One patient in the control group who did not have a history of COVID-19 died due to intracranial hemorrhage and hematoma postoperatively. In the postoperative ICU follow-up, one patient in the COVID-19 group and five patients in the control group had prolonged ICU and hospital stays. The reason for the extended stay of the patient in the COVID-19 group was respiratory-related; however, the patient also had advanced COPD. All other patients with prolonged stays were discharged from surgery under high-dose inotropes and IABP support. None of the other patients in the study developed comorbidities related to respiratory causes. Therefore, our findings suggest that, regardless of COVID-19 history, CABG with CPB support can be performed with promising results in terms of early outcomes and in-hospital mortality.

## Conclusion

The primary clinical concern beyond the inflammatory response triggered by cardiopulmonary bypass (CPB) support during coronary artery bypass grafting (CABG) is that prior COVID-19 infection may be associated with coagulopathy, endothelial injury, and vasculitis, all of which could contribute to postoperative graft vasculopathy and thromboembolic events. In our study, a history of mild COVID-19 infection did not appear to adversely affect coagulation parameters, vascular graft pathology, or early postoperative outcomes, including thromboembolic complications. Furthermore, despite previous reports suggesting that CPB might worsen outcomes following COVID-19, our findings indicate that CABG with CPB support can be performed safely in patients with a history of mild COVID-19, without a significant increase in early postoperative complications or in-hospital mortality.

Based on these findings, undergoing CABG surgery after a four-week recovery period appears to be safe for patients with a history of mild COVID-19, at least in terms of early postoperative vascular outcomes. However, these results should be interpreted with caution given the limitations of the study.

### Limitations and Suggestions for Future Research

Several limitations of this study must be acknowledged. First, the study population included only patients who had experienced mild or asymptomatic COVID-19 infections. As such, the findings cannot be generalized to patients who suffered moderate or severe forms of COVID-19, who may have ongoing systemic or vascular effects that could impact surgical outcomes. Second, the study focused solely on early postoperative outcomes and in-hospital mortality. Long-term follow-up data, including assessments of graft patency, thromboembolic complications, and survival, were not available. Therefore, it remains uncertain whether subtle vascular or endothelial injuries related to prior COVID-19 infection may manifest in adverse long-term outcomes. Third, although the study was conducted prospectively with consecutive enrollment, the sample size was relatively small, which may limit the statistical power to detect less common complications. Larger, multicenter studies would be valuable to confirm these findings across broader populations. Fourth, the uniform application of a minimum four-week waiting period post-COVID-19 before surgery was a strength in terms of standardization; however, the optimal timing for surgery following COVID-19 infection remains unclear and may vary depending on individual patient factors and the severity of the infection.

Future research should include larger cohorts, patients with a broader range of COVID-19 severities, and longer follow-up periods to better assess the potential long-term vascular consequences of prior COVID-19 infection in patients undergoing CABG surgery. Incorporating detailed vascular imaging, biomarker evaluation, and functional testing would also enhance understanding of potential subclinical impacts.

## Data Availability

The corresponding author can be contacted to obtain the anonymous dataset used and/or analyzed during the current study upon reasonable request.

## Declarations

### Research Involving Human and Animal Participants

Our study was conducted after the approval of the Local Clinical Research Ethics and Approval Committee (Numbered E-83045809-604.01.02-104678). Every human participant provided their informed consents in terms of involving the study. Our study was conducted in accordance with the Declaration of Helsinki.
